# What Killed the Woolly Mammoth?

**DOI:** 10.1371/journal.pbio.0060099

**Published:** 2008-04-01

**Authors:** Caitlin Sedwick

Forty-two thousand years ago, during the last glacial advance of the Pleistocene epoch, woolly mammoths thundered across the frozen steppes of the Eurasian continent. The huge beasts thrived on the arid tundra of the last ice age, having adapted to temperatures that would chill the toes off any hairless ape. Yet, by the middle of the Holocene epoch, 6,000 years ago, the glaciers had retreated and the Eurasian woolly mammoth was on the verge of extinction. They were ultimately done in, say David Nogués-Bravo and colleagues, by climate change—with a helping hand from humans.

Ever since Mikhail Adams retrieved the first fossilized woolly mammoth remains from Russia in 1806, scientists have debated what happened to this ancient relative of the Asian elephant (see doi:10.1371/journal.pbio.0040074). Did they die out because their habitat disappeared, as the planet's climate warmed and vegetation and precipitation patterns changed? Or did human interlopers on the Eurasian plains hunt them to extinction? It's a difficult question to untangle, because the retreat of the glaciers at the end of the Pleistocene changed the animals' habitat but also allowed primitive human bands to migrate north from southern Eurasia, likely hunting whatever game they encountered during their journey. While previous attempts to explain what happened to the mammoths were largely descriptive inferences based on available data, Nogués-Bravo et al. used the data to generate quantitative estimates of the interplay between the mammoth's disappearing range and warming versus hunting.

To examine the likely factors contributing to the mammoth's demise, the authors performed quantitative modeling of the climatic conditions inhabited by the mammoth in several periods of the last ice age. Their model related the fossil record—showing the distribution and age of mammoth remains—with simulated maps of the mean highest temperature, the mean lowest temperature, and average rainfall conditions on the Eurasian supercontinent for three time points during the last glacial advance in the Pleistocene (42,000, 30,000, and 21,000 years ago) and to a point in the previous interglacial middle of the Holocene (6,000 years ago). Next, they aligned their climatic models on the Eurasian supercontinent 126,000 years ago (the previous time the planet had warmed between glacial advances). Together, these data allowed the group to estimate the characteristics and extent of the animals' favored habitat at the various time points studied.

**Figure:**
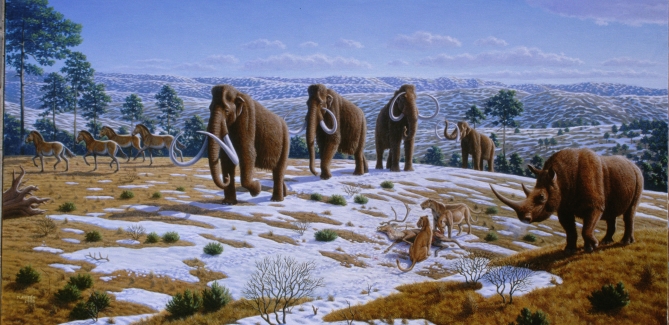
Woolly mammoths were driven to extinction by climate change and human impacts. (Credit: Mauricio Anton)

The authors' findings demonstrate that mammoths experienced a catastrophic loss of habitat: as the last glaciers retreated and the planet warmed, 90% of the animals' former habitat disappeared. Prime mammoth habitat progressively shrank from 7.7 million square kilometers 42,000 years ago (in the midst of the last glacial advance) until just 0.8 million square kilometers remained 6,000 years ago. The animals were restricted to isolated tracts spotted across Eurasia and tiny patches squeezed up against the northern coastal edges.

Although the near obliteration of their habitat would have placed great pressure on the species, the situation appeared even more dire during the previous glacial retreat 126,000 years ago, when only 0.3 million square kilometers of prime habitat existed. At that time, the species probably teetered on the brink of extinction, as geographically isolated groups experienced declines in genetic diversity and fitness. Even so, the mammoths had managed to survive that crucible. What was different about the Holocene? The remaining mammoth herds faced a foe that hadn't existed 126,000 years ago: human hunters.

Humans evolved to their modern form during the Pleistocene and migrated north with the final retreat of the glaciers, hunting mammoths as they advanced. By the middle of the Holocene, mammoth populations were so vulnerable that it would not have taken much hunting pressure to push them to extinction. Under the authors' most optimistic estimates of mammoth population size and density, if each human killed just one mammoth every 3 years, the species would go extinct. More pessimistic estimates of mammoth populations suggest that the loss of as few as one mammoth every 200 years (per human in its territory) might have sealed the animals' fate.

Other evidence may yet be uncovered that would support the authors' contention that mammoth populations made vulnerable by climate change were finished off by human hunting. For example, the authors' habitat models suggest new areas on the Eurasian continent where mammoth fossils may be found. Expeditions to these locations can determine whether mammoth populations lived there—and provide more evidence to help researchers continue the shift from qualitative to quantitative interpretations of the data.

